# Uncovering Gene Regulatory Networks from Time-Series Microarray Data with Variational Bayesian Structural Expectation Maximization

**DOI:** 10.1155/2007/71312

**Published:** 2007-06-27

**Authors:** Isabel Tienda Luna, Yufei Huang, Yufang Yin, Diego P Ruiz Padillo, M Carmen Carrion Perez

**Affiliations:** 1 Department of Applied Physics, University of Granada, Granada 18071, Spain; 2 Department of Electrical and Computer Engineering, University of Texas at San Antonio (UTSA), San Antonio, TX 78249-0669, USA

## Abstract

We investigate in this paper reverse engineering of gene regulatory networks from time-series microarray data. We apply dynamic Bayesian networks (DBNs) for modeling cell cycle regulations. In developing a network inference algorithm, we focus on soft solutions that can provide a posteriori probability (APP) of network topology. In particular, we propose a variational Bayesian structural expectation maximization algorithm that can learn the posterior distribution of the network model parameters and topology jointly. We also show how the obtained APPs of the network topology can be used in a Bayesian data integration strategy to integrate two different microarray data sets. The proposed VBSEM algorithm has been tested on yeast cell cycle data sets. To evaluate the confidence of the inferred networks, we apply a moving block bootstrap method. The inferred network is validated by comparing it to the KEGG pathway map.

## 

[1,2,3,4,5,6,7,8,9,10,11,12,13,14,15,16,17,18,19,20,21,22,23,24,25,26,27,28,29,30,31]
